# Maternal Social Loneliness Detection Using Passive Sensing Through Continuous Monitoring in Everyday Settings: Longitudinal Study

**DOI:** 10.2196/47950

**Published:** 2023-08-09

**Authors:** Fatemeh Sarhaddi, Iman Azimi, Hannakaisa Niela-Vilen, Anna Axelin, Pasi Liljeberg, Amir M Rahmani

**Affiliations:** 1 Department of Computing University of Turku Turku Finland; 2 Department of Computer Science University of California Irvine, CA United States; 3 Institute for Future Health University of California Irvine, CA United States; 4 Department of Nursing Science University of Turku Turku Finland; 5 Department of Obstetrics and Gynaecology Turku University Hospital Turku Finland; 6 Faculty of Medicine University of Turku Turku Finland; 7 School of Nursing University of California Irvine, CA United States

**Keywords:** health monitoring, internet of things, maternal loneliness, passive sensing, wearable device

## Abstract

**Background:**

Maternal loneliness is associated with adverse physical and mental health outcomes for both the mother and her child. Detecting maternal loneliness noninvasively through wearable devices and passive sensing provides opportunities to prevent or reduce the impact of loneliness on the health and well-being of the mother and her child.

**Objective:**

The aim of this study is to use objective health data collected passively by a wearable device to predict maternal (social) loneliness during pregnancy and the postpartum period and identify the important objective physiological parameters in loneliness detection.

**Methods:**

We conducted a longitudinal study using smartwatches to continuously collect physiological data from 31 women during pregnancy and the postpartum period. The participants completed the University of California, Los Angeles (UCLA) loneliness questionnaire in gestational week 36 and again at 12 weeks post partum. Responses to this questionnaire and background information of the participants were collected through our customized cross-platform mobile app. We leveraged participants’ smartwatch data from the 7 days before and the day of their completion of the UCLA questionnaire for loneliness prediction. We categorized the loneliness scores from the UCLA questionnaire as loneliness (scores≥12) and nonloneliness (scores<12). We developed decision tree and gradient-boosting models to predict loneliness. We evaluated the models by using leave-one-participant-out cross-validation. Moreover, we discussed the importance of extracted health parameters in our models for loneliness prediction.

**Results:**

The gradient boosting and decision tree models predicted maternal social loneliness with weighted *F*_1_-scores of 0.897 and 0.872, respectively. Our results also show that loneliness is highly associated with activity intensity and activity distribution during the day. In addition, resting heart rate (HR) and resting HR variability (HRV) were correlated with loneliness.

**Conclusions:**

Our results show the potential benefit and feasibility of using passive sensing with a smartwatch to predict maternal loneliness. Our developed machine learning models achieved a high *F*_1_-score for loneliness prediction. We also show that intensity of activity, activity pattern, and resting HR and HRV are good predictors of loneliness. These results indicate the intervention opportunities made available by wearable devices and predictive models to improve maternal well-being through early detection of loneliness.

## Introduction

Loneliness is a subjective, unpleasant feeling of mismatch between desired and perceived meaningful social relationships [1]. Loneliness can have adverse health consequences such as negative cardiovascular outcomes and mental health disorders [2] and even increase the risk of mortality [3]. In addition, loneliness is a global public health issue that is growing in modern society and has also slightly increased during the COVID-19 pandemic and its attendant social isolation [4-8].

Maternal loneliness during pregnancy and the postpartum period is associated with several health issues for the mother and her child. Various studies showed a positive correlation between loneliness and depression during pregnancy across countries [8-13]. In addition, maternal loneliness is associated with life dissatisfaction and pair-relationship dissatisfaction [12]. Other studies showed that loneliness was significantly associated with postpartum depression [14,15]. It was also shown that, in the COVID-19 pandemic, loneliness during pregnancy was associated with serious psychological distress [16], anxiety [17], cognitive distortion [13], higher level of perceived stress [9], and a lower level of social support [9,10]. Maternal loneliness increases the risk of respiratory tract infections in newborn babies [18]. The prediction or early detection of maternal loneliness could help avoid adverse consequences for the mother and her child through proper intervention.

Previous studies investigated loneliness during pregnancy and the postpartum period using observational methods based on self-reported measures, such as standard questionnaires [11] and interviews [19]. For example, Perzow et al [8] used self-report questionnaires to discern symptoms of depression and anxiety, loneliness, and COVID-19–related adverse health outcomes. In another study, Giurgescu et al [9] used web-based surveys to investigate the association between loneliness, depression, perceived stress, and social support during the COVID-19 pandemic in pregnant Black women. In another study [13], standard questionnaires were used to study the relationship between loneliness, depression, and cognitive distortion.

These studies investigated the associations between loneliness and various health issues, comparing the loneliness of people with health problems to the loneliness of those without health problems. However, they do not recommend or describe proactive services to predict or detect loneliness early on. In addition, subjective studies require participants’ engagement in answering the questionnaires or interview questions. Therefore, data collection is burdensome for pregnant women, especially in late pregnancy or during the postpartum period when they are occupied with a newborn baby and may find it difficult to remember and find time to answer questionnaires or engage in an interview.

Using wearable devices and smartphones for well-being and health care apps has been increasing rapidly in recent years. These devices enable continuous passive sensing of socio-behavioral data. However, few studies have used smartphones and wearable devices to *predict loneliness* using passive sensing [20,21]. The authors of one study [21] leveraged GPS and Bluetooth data gathered by participants’ smartphones to explore the association between momentary loneliness and companionship type in college students. In addition, another study [20] explored the sleep and physical activity data recorded on a wristband activity tracker, as well as the location, screen time, calls and SMS logs, and Bluetooth data of college students over the course of a semester and used this data to predict loneliness. Although these studies predicted loneliness by using wearable devices and passive sensing, they were limited to college students living on a university campus. Moreover, these studies did not use heart rate variability (HRV) features, even though it has been shown that loneliness is associated with lower resting HRV [22].

To the best of our knowledge, there is no study in the literature that has predicted maternal loneliness during pregnancy and the postpartum period on the basis of objective physiological data. The previously mentioned studies were limited to subjective data or performed on other population groups (ie, college students). Predicting maternal loneliness with the use of passive data sensing is beneficial to improving maternal and child well-being with minimal cost and effort required of mothers.

In this paper, we present a passive sensing method, enabled by a smartwatch, for loneliness prediction during late pregnancy and the postpartum period. The smartwatch collected heart rate (HR), HRV, physical activity, and sleep parameters. These physiological parameters were chosen due to the association of loneliness with lower resting HRV [22], decreased physical activity [23], and poor self-reported sleep quality [24,25]. We then developed 2 machine learning models—a decision tree and gradient boosting—to predict loneliness based on the objective data. Moreover, we investigated the importance of health parameters in loneliness prediction. In summary, the main contributions of this paper are as follows:

1. Presenting a passive sensing method, enabled by a smartwatch, for loneliness prediction during late pregnancy and the postpartum period.
2. Developing 2 machine learning models to predict loneliness during pregnancy and the postpartum period based on objective health data collected by a wearable device.
3. Investigating and discussing physiological parameters’ importance to maternal loneliness prediction.

## Methods

### Study Design

An observational longitudinal study was conducted in free-living conditions with a convenience sample of pregnant women in Southwest Finland. This study is part of a project using a wearable-based system to remotely monitor women’s physiological health parameters, including HR, HRV, sleep, and physical activity, during pregnancy and the postpartum period. This system used a smartwatch to collect objective health parameters and a cross-platform mobile app to collect subjective and background information. The remote maternal monitoring system was described and evaluated in a previous study [26]. As mentioned in [26], for privacy protection, we used the Secure Sockets Layer Application Programming Interface to provide secure communication between the cloud layer and our apps. We also used authentication for all of our apps, and we did not send or store any personal data on our cloud server.

### Participants and Recruitment

Pregnant women with singleton pregnancies were recruited at 12-15 gestational weeks for this study. The inclusion criteria were: (1) having the ability to understand the Finnish language; (2) being at least 18 years old; and (3) having an Android or iOS smartphone.

Recruitment was performed through maternity clinics or social media advertisements for 2 groups of pregnant women with different inclusion criteria from January 2019 to March 2020. The first group included women with a history of preterm birth (gestational weeks 22-36) or late miscarriage (gestational weeks 12-22). The second group consisted of women with a history of previous full-term, uncomplicated pregnancies and no pregnancy losses.

In scheduled face-to-face meetings with eligible volunteer pregnant women, the researchers informed the women about the study. Then, participants provided their written informed consent and received a smartwatch and the study instructions. The participants were asked to wear the smartwatch continuously during their pregnancies and for 3 months post partum. They also installed our customized cross-platform mobile app on their smartphones.

A total of 62 pregnant women were recruited for this study. Out of which 4 women withdrew from the study. We also excluded data from participants with a high amount of missing data (see *Data Sets and Machine Learning Models for Loneliness Prediction*). Thus, 31 pregnant women were included in this study. The participants’ background information is provided in Table 1.

**Table 1:** Participant background information (N=31).

Parameters			Values
Age (years), mean (SD)			32.9 (4.97)
BMI, mean (SD)			26.3 (8.6)
**Marital status, n (%)**			
	Married or cohabitation	30 (96.8)	
	Other	1 (3.2)	
**Work status, n (%)**			
	Working	26 (83.9)	
	Student	1 (3.2)	
	Unemployed	0 (0)	
	Other	4 (12.9)	
**Education, n (%)**			
	High school	11 (35.5)	
	College	7 (22.6)	
	University	13 (41.9)	
**Pregnancy planned, n (%)**			
	Yes	30 (96.8)	
	No	1 (3.2)	
Pregnancy week at recruitment (gestational week+day), mean (SD)			14+3 (1+4)
Pregnancy weeks at birth (gestational week+day), mean (SD)			39+2 (1+4)
**Mode of delivery, n (%)**			
	Vaginal	24 (77.4)	
	Cesarean	7 (22.6)	
Infant birth weight (g), mean (SD)			3542.4 (556.3)

### Data Collection

Data were collected from each participant by a Samsung Gear Sport smartwatch and by our customized cross-platform mobile app. The Samsung smartwatch included a photoplethysmography (PPG) sensor and an inertial measurement unit. It ran Tizen OS, which is an open-source operating system that enabled us to develop customized apps for the smartwatch. The smartwatch provided PPG signals, acceleration data, and gyroscope data. We developed customized apps for the smartwatch to collect sleep and physical activity data continuously and collect 12 minutes of PPG signal every other hour. The data were stored on the internal storage of the smartwatch. In addition, we developed a smartwatch app to transfer the collected data to our cloud server using Wi-Fi. The smartwatch, enabled by our apps, had sufficient battery life (ie, 2-3 days) for data collection [26].

Our customized cross-platform mobile app provided self-report questionnaires to the participants. To evaluate loneliness, we used the 12-item version of the revised University of California, Los Angeles (UCLA) Loneliness Scale questionnaire, consisting of questions about the factors of social and emotional loneliness [27]. Each factor was addressed by 6 questions, to which the answers had potential scores ranging from 6 to 24. Higher points indicated greater feelings of loneliness. We followed the participants from gestational weeks 12-15 throughout the pregnancy and until 3 months postpartum. The structured questionnaires were decided to be sent at week 36 to capture the situation at the end of the pregnancy (third trimester) and at 12 weeks postpartum to capture the situation after the birth, when the mother had already had some time to adapt to living with the newborn baby. We also collected background information through the mobile app.

### Data Sets and Machine Learning Models for Loneliness Prediction

**Figure 1:**
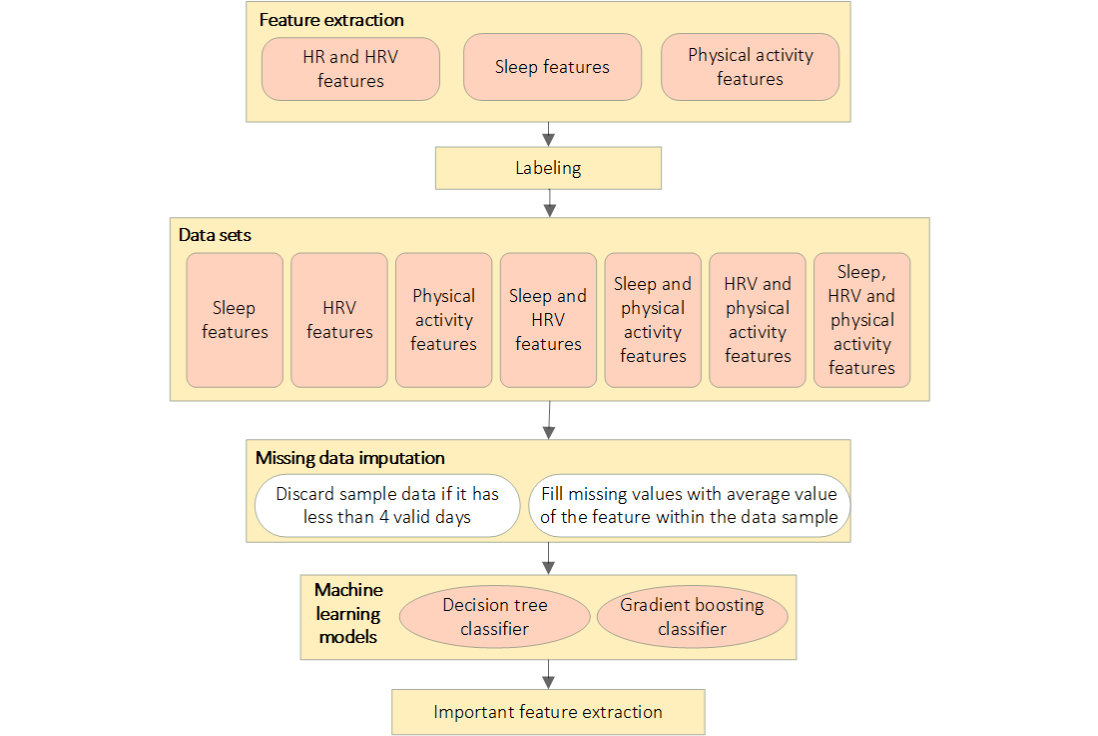
Machine learning pipeline. HR: heart rate; HRV: heart rate variability.

The collected data from the smartwatch were used to generate 7 data sets. Then, we developed 2 machine learning models and used these data sets to train and test our models. Finally, we investigated the important parameters for loneliness prediction. Our machine learning pipeline, of which an overview is shown in Figure 1, comprised the following processes:

1. Feature extraction
2. Data set creation and labeling
3. Missing data imputation
4. Training and testing the machine learning models (decision tree and gradient boosting) for different data sets
5. Investigating the important features of the 2 machine learning models for loneliness prediction

These steps are described in the following sections.

### Feature Extraction

We extracted HR and HRV, sleep, and physical activity data from the objective data collected by the smartwatch.

### HR and HRV Features

We used PPG signals to extract HR and HRV features. The smartwatch collected 12 minutes of PPG signals every other hour with a sampling frequency of 20 Hz (as described in a previous study [26]). Based on the duration of PPG recordings, we used short-term HRV analysis, including 5-minute windows of PPG signals, for HRV extraction [28,29]. Our HR and HRV extraction pipeline consisted of 3 steps.

#### Reliable Signal Detection

PPG signals are prone to noise, such as motion artifacts. Therefore, unreliable signals had to be detected and discarded. Therefore, we used the PPG signal quality assessment method introduced in [30], which used a one-class support vector machine (SVM) classifier to distinguish reliable and unreliable signals [30]. This classifier was trained using several morphological features of the PPG signals, such as the correlation between the cardiac cycles. Then, we leveraged the trained model to detect and subsequently discard unreliable PPG signals.

#### Peak Detection and Interbeat Interval Extraction

We used a bandpass filter with cutoff frequencies of 0.7 Hz and 3.5 Hz to filter out noise outside the human heart rate range. We used a moving average-based peak detection method with adaptive thresholds to detect peaks and extract interbeat intervals (IBIs). Then, we used error detection methods to remove false peaks and their corresponding IBIs. To this end, too-large or too-small IBIs were removed based on the other IBIs in the same window of the signals. The peak detection and IBI extraction method was implemented using HeartPy library [31] in Python.

#### Resting HR and HRV Extraction

We extracted resting HR (when the HR is lowest during sleep) and its corresponding HRV parameters using detected peaks and extracted IBIs. HR is calculated as the number of peaks per minute. We used normal IBIs to extract HRV parameters that could be reliably extracted at the sampling frequency of collected PPG signals (20 Hz) [32]. The extracted HRV parameters were average normal IBIs (AVNN), root-mean-square of the successive differences (RMSSD), SD of IBIs (SDNN), power in the low-frequency range (LF), power in the high-frequency range (HF), and LF to HF ratio (LF/HF).

### Sleep Features

Using the smartwatch, we recorded total sleep time (TST), sleep fragmentation, wake after sleep onset (WASO), and average hand movement during sleep, as described in a previous study [33]. We also added a sleep quality indicator showing WASO of ≤20 minutes [34] and a sufficient sleep parameter, which showed TST between 7 and 8.5 hours [35].

### Physical Activity Features

The smartwatch captured several physical activity parameters at a granularity of 10 minutes. By aggregating the smartwatch’s activity parameters during participants’ awake time, we extracted daily step counts, walking steps, running steps, distance, activity duration, and activity intensity. We then calculated the sedentary time as awake time without walking and running activities. We also added a sufficient activity indicator to show daily step counts above 7000. Finally, we extracted statistical parameters from the distribution of step counts and duration of activity during the day, based on the hourly data. The statistical parameters of hourly activity distribution were mean, minimum, median, maximum, SD, IQR, range, skewness, kurtosis, and root-mean-square. We selected the most relevant physical activity features to be used in our models. Table 2 shows the summary of extracted features.

**Table 2:** Summary of extracted features.

Parameters		Unit	Description
**HR^a^ and HRV^b^ features**			
	HR	bpm	Number of heartbeats per minute
	AVNN^c^	ms	Average of normal IBIs^d^
	SDNN^e^	ms	SD of normal IBIs
	RMSSD^f^	ms	Square root of the mean of the sum of the squares of differences between adjacent normal IBIs
	LF^g^	ms^2^	Power in low-frequency range (0.04-0.15 Hz)
	HF^h^	ms^2^	Power in high-frequency range (0.15-0.4 Hz)
	LF/HF	N/A^i^	Ratio of LF to HF
**Sleep features**			
	TST^j^	Min	Total sleep time
	Sleep fragmentation	N/A	Number of sleep interruptions during the night
	WASO^k^	Min	Wake after sleep onset
	Average hand movement	N/A	Average intensity of hand movement during sleep, provided by the smartwatch
	Sleep quality indicator	N/A	Indicate whether WASO is of ≤20 minutes or not
	Sufficient sleep parameter	N/A	Indicate whether TST is between 7 and 8.5 hours
**Physical activity features**			
	Step counts	N/A	Total step counts during the day
	Walking steps	N/A	Total steps walked during the day
	Running steps	N/A	Number of running steps during the day
	Distance	m	The distance traveled during the day
	Activity duration	Min	Duration of activity during the day
	Activity intensity	N/A	Intensity of activity based on the calories burned provided by the watch
	Sedentary time	Min	Awake time without activity
	Sufficient activity indicator	N/A	Indicate whether activity was sufficient (step count above 7000) or not
	Statistical features from the distribution of step counts	N/A	Mean, minimum, median, maximum, SD, IQR, range, skewness, kurtosis, and root mean square of hourly step counts
	Statistical features from the distribution of activity duration	N/A	Mean, minimum, median, maximum, SD, IQR, range, skewness, kurtosis, and root mean square of hourly activity duration

^a^HR: heart rate.

^b^HRV:heart rate variability.

^c^AVNN: average normal IBIs.

^d^IBI: interbeat interval.

^e^SDNN: SDs of IBIs.

^f^RMSSD: root-mean-square of the successive differences.

^g^LF: low-frequency range.

^h^HF: high-frequency range.

^i^N/A: not applicable.

^j^TST: total sleep time.

^k^WASO: wake after sleep onset.

### Data Set Creation and Labeling

We generated 7 data sets using different combinations of HR and HRV, sleep, and physical activity features extracted from the smartwatch data. These data sets were used to train and test our developed machine learning models.

Labeling was performed using UCLA scores. We only considered UCLA social loneliness factors and ignored the UCLA emotional scores since we had few participants with high emotional scores. We used binary classification for loneliness prediction and considered UCLA social score of ≥12 as 1 (loneliness) and UCLA social score of <12 as 0 (no loneliness) [27]. We used UCLA participants’ responses at gestational week 36 and week 12 after delivery as labels in our data set. We combined the data from both time points as the data set was small.

The UCLA questions ask respondents to consider their feelings over the previous week (7 days) [27]. Therefore, for each sample in our data sets, we included data from 7 days before and the day of answering the UCLA questionnaire (ie, 8 days of data).

### Missing Data Imputation

We added a data sample that contained 8 days of data and a loneliness label from one participant in the data set if the data sample had at least 4 valid days. A valid day was defined as a day in which the participant wore the smartwatch for at least 10 hours during waking hours and in which the watch collected valid sleep data [36-40]. The samples with fewer than 4 valid days were discarded due to the high proportion of missing data. In addition, we used the average values of each feature in one data sample to fill in the missing values in that data sample. Table 3 represents the wear time per day in our data set.

**Table 3:** Wear time per day in our data sets.

Day	Wear time (minutes), mean (SD)
1	1170 (210)
2	1255 (150)
3	1150 (200)
4	1220 (170)
5	1240 (150)
6	1320 (100)
7	1270 (145)
8	1310 (130)

We added 39 data samples from 31 participants (8 participants had 2 data samples) to our data sets. The data from other participants were excluded due to the high ratio of missing data (ie, less than 4 valid days in the week before and the day of answering the UCLA questionnaire). The missing data resulted from different technical and practical issues during monitoring. For example, some participants had preterm births before gestational week 36. Many wore the watch for an insufficient amount of time. Some participants removed the smartwatch’s customized app by resetting the watch.

### Machine Learning Models

We developed decision tree and gradient boosting models for predicting loneliness and investigated the importance of features for loneliness prediction.

A decision tree classifier is a simple, flexible, robust, and easy to interpret method, which is well suited to complex ecological data [41,42]. This model has a tree-like structure that includes internal nodes and leaves. Each internal node splits the data based on one feature. The features are selected based on the Gini index, which represents the purity of classification. Each leaf node shows the class label.

Gradient boosting is another machine learning method that can be used for classification and regression. It is an ensemble of weak prediction models, and in each step of training, it adds a new estimator to improve the results. This model performs well on noisy data and outperforms most common machine learning models [43].

The decision tree method was chosen as it is fast and simple, and a specific feature’s importance can be easily understood from the tree structure. In addition, the gradient boosting model was chosen as it performed well in prediction with nonlinear decision boundaries and has produced good results in similar studies [20].

### Model Evaluations

To evaluate the models, we first used feature selection on physical activity features to avoid overfitting. We used the recursive feature elimination (RFE) method, which recursively eliminated the least important features. Then, we investigated the performance of the predictive machine learning models using the leave-one-participant-out cross-validation method. Therefore, we used one participant’s data for validation, and other participants' data were used for training. The evaluation was performed for all participants, and the average performance was reported.

The following machine learning measures were used for performance evaluation:

- Precision: percentage of predicted samples that actually belonged to a class
- Recall: percentage of correctly predicted samples per class
- *F*_1_-score: harmonic mean of precision and recall per class
- Weighted *F*_1_-score: weighted average of *F*_1_-scores
- Sensitivity: percentage of lonely participants correctly detected
- Specificity: percentage of nonlonely participants correctly detected
- AUC (area under the curve) score: area under the receiver operating characteristic (ROC) curve

Finally, we investigated important features for each model, for example, the features that contribute most to each model. The machine learning models, feature selection, model evaluation, and important feature investigation were implemented using the scikit-learn library in Python [44].

### Ethics Approval

This study received ethical approval from the Ethics Committee of the Hospital District of Southwest Finland (approval number DNRo: 1/1801/2018). Written informed consent was obtained from all participants.

## Results

In this section, we present the performance of our predictive models in terms of precision, recall, *F*_1_-score, and weighted *F*_1_-scores. We also discuss the importance of features in loneliness prediction.

### Loneliness Prediction

**Figure 2:**
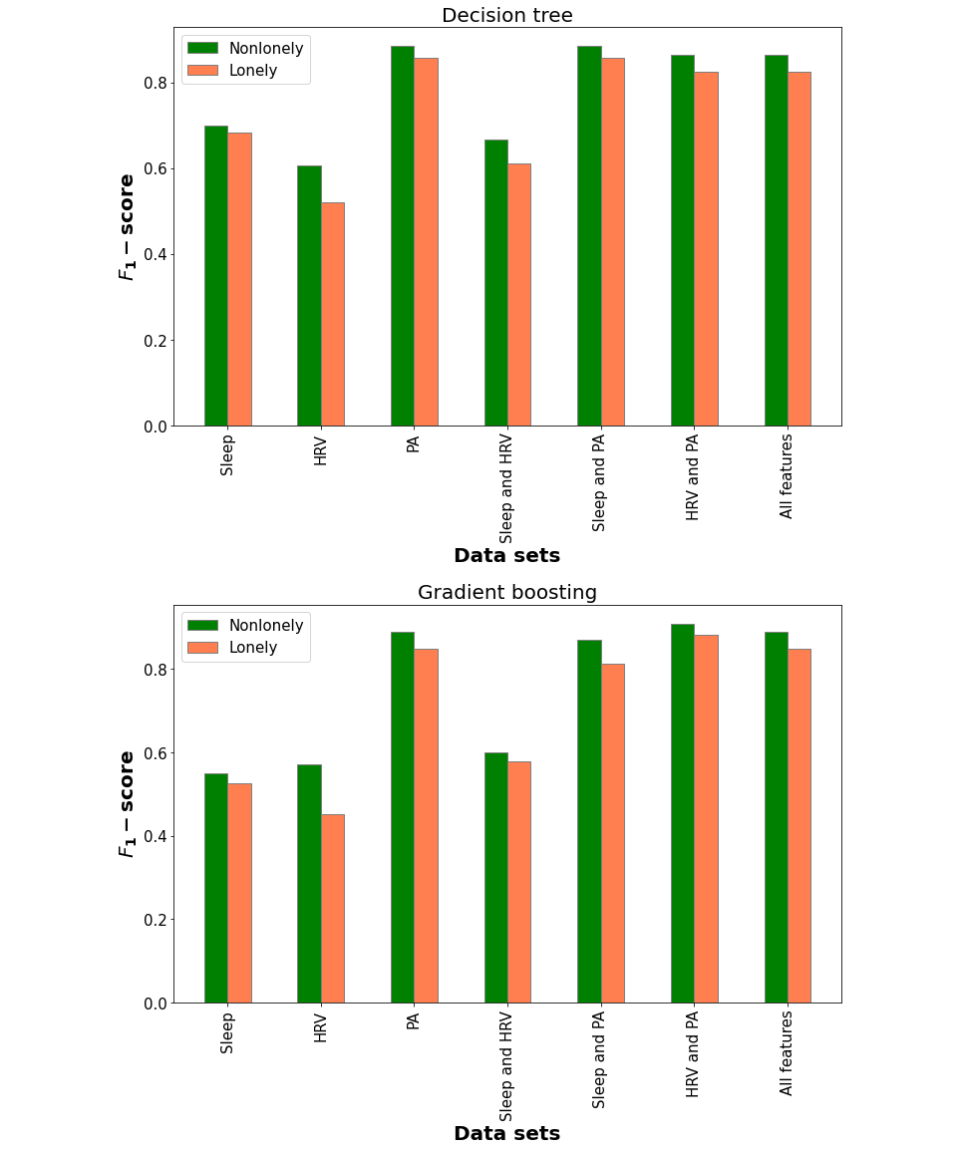
F1-scores of decision tree and gradient boosting for different data sets. HRV: heart rate variability; PA: physical activity.

The prediction results of the predictive models for different data sets are summarized in Table 4. The data sets contain HRV features, sleep features, physical activity features, and different combinations of these feature sets. Figure 2 illustrates the *F*_1_-scores of the decision tree and gradient boosting for different data sets.

**Figure 3:**
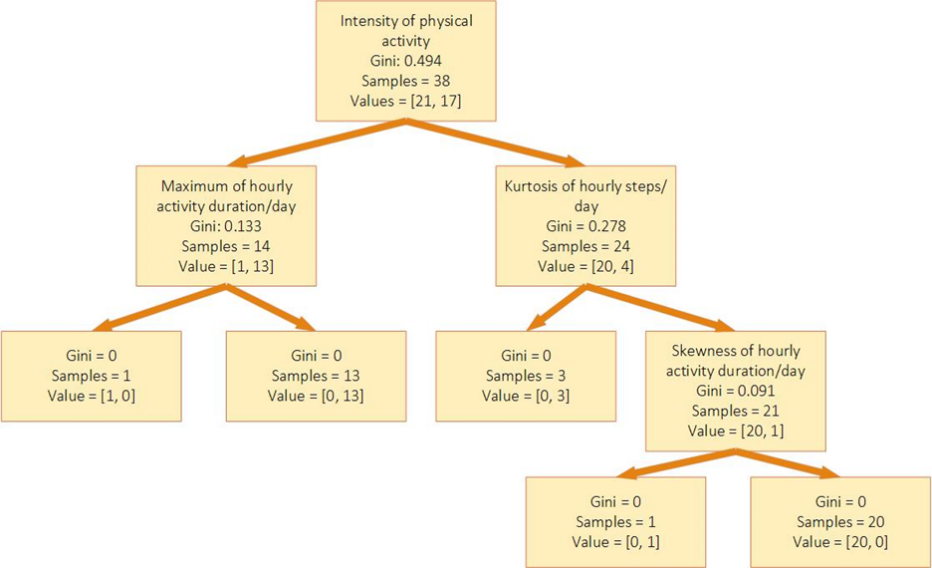
Features used in decision tree model for physical activity data set.

The decision tree model achieved the best performance on data sets that contained physical activity features (physical activity features, physical activity and HRV features, physical activity and sleep features, or all the features). The results show that physical activity features had the highest impact on the prediction results for the decision tree model. Moreover, the specificity of the models for all the data sets containing physical activity features is 0.864 which shows that the decision tree can detect nonlonely persons correctly with high accuracy. Furthermore, the sensitivity of the decision tree for physical activity features or all features is 0.882, which shows that the model performance is very high in correctly detecting loneliness. Figure 3 shows the decision tree model for the physical activity features data set and the features used in the loneliness predictions.

**Table 4:** Per class precision, recall and F1-score, weighted F1-score, sensitivity, specificity, and area under the curve (AUC) score performance measures for the predictive models. Data sets with weighted an F1-score of >0.8 are shown in italics.

Models and data sets		Precision			Recall			*F*_1_-score			Weighted *F*_1_-score		Sensitivity		Specificity		AUC
		Nonlonely	Loneliness	Nonlonely		Loneliness	Nonlonely		Loneliness								
**Decision tree**																	
	Sleep	0.778	0.619	0.636		0.765	0.7		0.684	0.693		0.765		0.636		0.7	
	HRV^a^	0.6	0.528	0.614		0.514	0.607		0.521	0.567		0.513		0.614		0.563	
	PA^b^	*0* *.905*	*0.833*	*0.864*		*0.882*	*0.884*		*0.857*	*0.872*		*0.882*		*0.864*		*0.873*	
	Sleep and HRV	0.7	0.579	0.636		0.647	0.667		0.611	0.642		0.647		0.636		0.642	
	Sleep and PA	*0.905*	*0.833*	*0.864*		*0.882*	*0.884*		*0.857*	*0.872*		*0.882*		*0.864*		*0.873*	
	HRV and PA	*0.864*	*0.824*	*0.864*		*0.824*	*0.864*		*0.824*	*0.846*		*0.824*		*0.864*		*0.844*	
	All	*0.864*	*0.824*	*0.864*		*0.824*	*0.864*		*0.824*	*0.846*		*0.824*		*0.864*		*0.844*	
**Gradient boosting**																	
	Sleep	0.611	0.476	0.5		0.588	0.55		0.526	0.540		0.588		0.5		0.544	
	HRV	0.553	0.471	0.591		0.432	0.571		0.451	0.516		0.432		0.591		0.512	
	PA	*0.87*	*0.875*	*0.909*		*0.824*	*0.889*		*0.848*	*0.871*		*0.823*		*0.909*		*0.866*	
	Sleep and HRV	0.667	0.524	0.545		0.647	0.6		0.579	0.591		0.647		0.545		0.590	
	Sleep and PA	*0.833*	*0.867*	*0.909*		*0.765*	*0.87*		*0.812*	*0.845*		*0.765*		*0.909*		*0.837*	
	HRV and PA	*0.909*	*0.882*	*0.909*		*0.882*	*0.909*		*0.882*	*0.897*		*0.882*		*0.909*		*0.896*	
	All	*0.87*	*0.875*	*0.909*		*0.824*	*0.889*		*0.848*	*0.871*		*0.823*		*0.909*		*0.866*	

^a^HRV: heart rate variability.

^b^PA: physical activity.

As did the decision tree model, the gradient boosting model had better classification results for data sets containing physical activity than for other data sets. Moreover, gradient boosting performed better on the data set of physical activity features than it did on the data set of physical activity and sleep features. The model’s performance is the same for all features and physical activity features. However, the gradient boosting model achieved higher performance on the data set containing both physical activity and HRV features than it did on any other data set.

The gradient boosting model can correctly detect nonlonely persons with more than 90% accuracy on data sets that contain physical activity features. Moreover, this model achieves a performance higher than 88% in detecting lonely participants. The results show that adding sleep features can negatively affect the performance of gradient boosting in correctly detecting lonely people but has no effect on detecting nonlonely people.

### Feature Importance in Loneliness Prediction

We investigated the importance of the features in the decision tree and the gradient boosting models on data sets that achieved a weighted *F*_1_-score higher than 80%. For the decision tree, in the 4 models with the highest *F*_1_-score, the most important features were the intensity of activity and kurtosis of the steps during the day (based on hourly data). Other features that contributed to the prediction were resting SDNN, LF, LF/HF, maximum, median, and mean duration of activity during the day. For gradient boosting, same as the decision tree, the most important features were also the intensity of activity and kurtosis of the step count. Other features include several distribution parameters of total steps and duration during the day, such as kurtosis, maximum, average, IQR, SD, range, and root-mean-square, sedentary time, and HRV features (including resting HR, LF/HF, SDNN, and AVNN) also contribute in the gradient boosting model prediction.

The most frequently selected features with high importance in these models show their significant impact on prediction. Therefore, the results show that intensity of activity and activity distribution during the day have the highest association with and effect on loneliness.

## Discussion

### Principal Findings

In this study, we developed 2 predictive models—decision tree and gradient boosting—to predict loneliness during late pregnancy and the postpartum period by using physiological data collected by a smartwatch. The models used 8 days of data collected passively by a smartwatch to predict maternal social loneliness. The gradient boosting and decision tree models achieved weighted *F*_1_-scores of 0.897 and 0.872, respectively. Moreover, both models achieve the same sensitivity. However, gradient boosting has higher specificity than the decision tree model, indicating that gradient boosting performs better at correctly detecting nonlonely people. These results show the feasibility of predicting maternal loneliness during pregnancy and the postpartum period by passive sensing using wearable devices.

In addition, we investigated the importance of sleep, resting HR and HRV, and physical activity collected by the smartwatch for loneliness prediction. Our results show that physical activity, patterns of activity during the day, and resting HR and HRV are the most important predictors of loneliness. The decision tree results show that having high or intensive activity levels (ie, when most of a participant’s daily steps happen within a short period of time) can be a good sign of nonloneliness. On the other hand, having less intensive activity levels and a low resting HRV when most of a participant’s activity takes place before evening can be a predictor of loneliness.

This finding about the association between low physical activity and increased loneliness is very important for maternity care. It is well known that women’s levels of physical activity decrease as pregnancy proceeds [39]. By contrast, high levels of prenatal activity and exercise are associated with lower pregnancy-related and obstetric complications as well as a higher health-related quality of life [45-47]. Though a low level of physical activity may, in itself, be a risk for many adverse outcomes, it could also be a sign of loneliness and thereby further increase negative health consequences. Therefore, health care professionals should encourage pregnant women to be physically active but, simultaneously, should be attentive to the signs of loneliness so that they are able to support pregnant women individually and, by implication, promote the health of both the mother and her fetus or infant.

### Comparison With Previous Studies

To the best of our knowledge, this is the first study predicting loneliness during pregnancy and the postpartum period based on objective health parameters. Previous work usually considered college students or young adults [20,21,48], and older people [49].

Badal et al [49] used natural language processing methods to predict loneliness in older people. Our results show higher precision, recall, and *F*_1_-scores than their results for quantitative loneliness prediction. Moreover, their method required a semistructured interview. However, our method passively collects data and requires no further effort from participants. In another study [21], researchers used GPS and Bluetooth data gathered by participants’ smartphones as well as ecological momentary assessment surveys collecting real-time self-report information about companionship types and social interactions. Their models can predict self-report loneliness with an average AUC equal to 0.74. In contrast, our models have better performance, and we used a standard UCLA questionnaire for labeling. Moreover, Doryab et al [20] predicted loneliness for college students with an accuracy of 80.2%, based on data collected from a smartphone and a wearable device. Our results for pregnant women achieved higher performance than their work did. Moreover, their model requires the use of more information from participants (such as the phone numbers of close friends or family members, used to assess calls to close contacts), which raises privacy concerns. However, our work only used physiological parameters.

In addition, some studies investigated important features in loneliness prediction. Wang et al [48] showed that daily activity duration, traveled distance, and activity duration in the evening are negatively correlated with loneliness in college students. Other studies also showed a negative correlation between loneliness, duration of activity, total movements, and step counts [20,50]. This is in alignment with our results, which show physical activity features to be the most important factors in loneliness predictions. Ben-Zeev et al [51] reported that loneliness was not associated with sleep duration, a result confirmed by our study’s finding regarding participants’ sleep parameters.

### Limitations and Future Work

We have 39 valid data samples with which to train and test our predictive models. The data set is small, but the findings provided valuable insights, showing potential in this research direction. The outcomes serve as a starting point, indicating the viability of such loneliness detection methods and showing future directions to pursue larger-scale investigations to validate and expand upon our initial findings. In the future, we will need to test our predictive models with more data in order to generalize the results. In addition, the participants in this study were healthy. Therefore, the predictive models’ validity is limited to a healthy population. In the future, we should consider including participants with diagnosed health problems.

We used data from late pregnancy (gestational week 36) and 12 weeks postpartum for loneliness prediction. However, it is known that physiological health parameters such as HRV and physical activity change during pregnancy and the postpartum period [52,53], for example, physical activity decreases during pregnancy. Generalizing our predictive models to the whole pregnancy requires using data from additional weeks during pregnancy and the postpartum period. Moreover, we combine the data from pregnancy and postpartum to train our data set. In the future, with a larger data set, we should investigate pregnancy and postpartum separately and examine the discrepancy between the 2 stages of maternal loneliness.

We used predictive models for detecting loneliness, which predict loneliness based on objective health data. However, these models lack the causality effects between loneliness and objective parameters. In the future, we should use causal inference methods to investigate the cause-and-effect relations between the parameters.

### Conclusions

In this paper, we present predictive machine learning models for loneliness prediction during pregnancy and the postpartum period. Using HRV, sleep, and physical activity data collected by smartwatches, our presented predictive models achieved high *F*_1_-scores. Our findings illustrate the potential benefit and feasibility of predicting loneliness during pregnancy by using objective data collected passively through a smartwatch. In addition, our findings provide insight into which physiological parameters are associated with loneliness during late pregnancy and the postpartum period. Using passive sensing and predictive models to predict and detect loneliness can support the creation of interventions based on prediction outcomes and thereby effectively improve maternal and infant well-being and prevent adverse health outcomes related to loneliness.

## Abbreviations

AUC: area under the curve
AVNN: average normal IBIs
HF: high-frequency range
HR: heart rate
HRV: heart rate variability
IBI: interbeat interval
LF: low-frequency range
PPG: photoplethysmography
RFE: recursive feature elimination
RMSSD: root-mean-square of the successive differences
ROC: receiver operating characteristic
SDNN: SD of IBIs
SVM: support vector machine
TST: total sleep time
UCLA: University of California, Los Angeles
WASO: wake after sleep onset

## Data Availability

The data used in this study include sensitive health information, and the informed consent signed by the participants does not allow the data to be made publicly available due to ethical restrictions. According to the current approval by the Ethics Committee of the Hospital District of Southwest Finland, the participants gave permission to use the collected data only for the purpose described in the consent. Data requests may be subject to individual consent and ethics committee approval. Researchers wishing to use the data should contact the Ethics Committee of the Hospital District of Southwest Finland; contact details: Tyks U-hospital, Kiinamyllynkatu 4-8, UB3, PO Box 52, FI-20521 TURKU, Finland; e-mail: eettinen.toimikunta@tyks.fi. We recommend first contacting the PI of the research project, associate professor Anna Axelin; contact details: University of Turku, Department of Nursing Science, 20014 University of Turku, Finland; e-mail: anmaax@utu.fi.
